# Fasting as a metabolic strategy for cancer prevention: emerging evidence and future direction

**DOI:** 10.1097/MS9.0000000000004597

**Published:** 2025-12-16

**Authors:** Aliya Noor, Zahra Batool, Areeba Shaheen, Aiman Shahzad, Ali Rohan, Kamil Ahmad Kamil

**Affiliations:** a Department of Medicine, King Edward Medical University, Lahore, Punjab, Pakistan; b Department of Medicine, University College of Medicine and Dentistry, University of Lahore, Punjab, Pakistan; c Department of Medicine, Internal medicine department, Mirwais Regional Hospital, Kandahar, Afghanistan

*Dear Editor,*

Cancer is one of the greatest public health challenges worldwide and is responsible for nearly 20 million new cases and 9.7 million deaths annually. Moreover, the prevalence of cancer cases and deaths is expected to increase by 77 and 90% in 2025, respectively^[1]^. With this emergence of worldwide incidence and prevalence, there is an increased interest in preventive measures that are affordable, easily available, and biologically based. Fasting and energy restriction programs are among the new strategies that have attracted considerable interest in terms of their ability to decrease risk factors for cancer^[2]^. An increasing body of mechanistic knowledge and encouraging preclinical evidence points to the possibility of beneficial results of fasting as a cancer preventive measure when safely and properly practiced^[3]^. This letter to the editor adheres to the TITAN guideline on the need for transparency in AI use in health care^[4]^.

This concept is grounded on the premise of extensive preclinical research in animal subjects. A comprehensive systematic review and meta-analysis study of animal studies revealed that caloric restriction was a potent suppressive factor on the development of tumors in a series of models. Impressive results were reported where 90% of the included studies demonstrated anticarcinogenic effects^[5]^. These results are all indicative of a fasting-induced metabolic state that is not conducive to cancer formation. The primary protective effects of fasting include reduction in circulating glucose, insulin and insulin like growth factor-1 (IGF-1) which are the key mediators of cell growth and proliferation^[6]^. Therefore, the lower levels of these growth signals decline the proliferation propensity and interfere with the pathways utilized during the initial stages of malignant transformation.

Another mechanism by which fasting enhances protection against cancer development is through activation of several cellular mechanisms. These involve autophagy, enhancement of DNA repair, protection from oxidative stress, and inhibition of systemic inflammation. All these mechanisms play a central role in ensuring genomic stability and barring the development of neoplasms^[6]^. Moreover, healthy cells respond to fasting by entering a stress resistant state. Precancerous or malignant cells on the other hand become more vulnerable to nutrient scarcity due to their high metabolic demands and impaired stress response capacity^[7]^. Fasting is also capable of supporting healthier body weight, decreasing visceral adiposity, and enhancing metabolic health, which helps in countering a number of long-standing risk factors of cancer^[8]^. These implicit disparate reactions constitute one of the most persuasive biological justifications to support fasting as one of the preventive measures.

The role of fasting in modulation of immune function also adds to its possible anticancer effects. Fasting has been reported to produce better immunosurveillance, greater cytotoxic lymphocyte response and reduction in the production of proinflammatory cytokines^[9]^. Fasting aids in the maintenance of a less favorable immunological microclimate against cancer formation. Preventative fasting is also in line with the wider public health requirements. Existing cancer prevention methods like pharmacological chemoprevention or special screening have been found to be expensive and hard to apply on a large scale. Fasting, on the contrary, is cheap, cross-cultural, and not dependent on high-level medical services.

In addition to these favorable aspects, responsible implementation is essential since fasting is not appropriate for everyone such as underweight individuals, elderly, pregnant, and those with chronic illnesses. It is also not recommended in patients who are receiving cancer treatment unless supervised^[10]^. Extensive or strenuous fasting without any form of supervision could cause nutritional deficiencies, muscle atrophy, and metabolite imbalance as the best fasting regimens are yet to be fixed^[11]^. Nevertheless, the facts in favor of the idea of fasting as a preventive therapy cannot be ignored. Combination of metabolic, cellular, and immunological advantage and safety profiles in preclinical human trials, fasting might become a useful, convenient, and non-pharmacological way to decrease cancer risk. In order to fully utilize its potential, we need to carry out large scale prospective trials to establish long-term impacts on cancer incidence. We should also create effective and safe fasting guidelines that should be based on the needs of various populations.

Conclusively, with the increased prevalence of cancer in the world^[1]^, all potential ways of preventing and treating cancer should undergo stringent pursuance. Fasting, in this context, comes out to be one of the possible and the least costly prevention strategies of cancer. It is necessary to develop formal fasting recommendations and increase the awareness of the general population of its potential to prevent cancer. Powerful clinical trials in humans should be conducted to adequately support the future preventive cancer strategies. When systematically pursued, fasting has the potential of becoming a radical preventive modality in the cancer prevention terrain that would transform how patients are treated in the future.
